# Parameter Estimation for the Basic Zirka-Moroz History-Dependent Hysteresis Model for Electrical Steels

**DOI:** 10.3390/ma18092104

**Published:** 2025-05-03

**Authors:** Martin Petrun, Ermin Rahmanović

**Affiliations:** Institute of Electrical Power Engineering, Faculty of Electrical Engineering and Computer Science, University of Maribor, 2000 Maribor, Slovenia; ermin.rahmanovic@um.si

**Keywords:** history-dependent hysteresis model, grain-oriented electrical steel, magnetic hysteresis, non-oriented electrical steel, parameter estimation, static hysteresis

## Abstract

History-dependent hysteresis models can potentially describe magnetization curves of all orders accurately. This property is essential for modeling magnetization and power loss in magnetic components subjected to distorted excitation waveforms, which result in complex magnetization patterns such as offset minor loops. The basic Zirka–Moroz history-dependent hysteresis model offers a good balance between the model’s complexity and accuracy. However, estimating the model’s parameters can be challenging. This research provides insight into the parameter estimation procedure for the discussed hysteresis model. Based on the measured first-order reversal curves, the fundamental two-step parameter estimation procedure was employed and analyzed for two non-oriented and one grain-oriented electrical steel types used widely in contemporary electric drives and electromagnetic devices. For each sample evaluated, two sets of parameters were estimated and compared to the reference parameters recommended for non-oriented electrical steels. The performed analysis is essential for gaining a comprehensive understanding of the capabilities, challenges, requirements, and limitations associated with estimating the parameters and performance of the analyzed model for specific electrical steel types.

## 1. Introduction

The existence of a large number and the ongoing development of new hysteresis models shows that none of the existing hysteresis models are universally applicable, where each of them has its advantages and disadvantages [1,2,3,4,5,6,7,8,9,10,11,12,13,14,15,16,17,18,19,20,21]. With the exponential increase in contemporary electric drives and electromagnetic devices supplied by power electronics, the requirements regarding the modeling of magnetization and power loss have been elevated. Power electronic supplies generally generate distorted excitation waveforms, which result in complex magnetization profiles within magnetic cores, including offset hysteresis loops, despite the fundamentally cyclic excitation [12,13,22,23,24,25,26]. Furthermore, adequate modeling of magnetization and power loss for novel soft magnetic materials is crucial for the evolution of emerging technologies, such as additive manufacturing [27].

One of the more popular hysteresis models in applied engineering is, e.g., the well-known Jiles–Atherton (JA) hysteresis model and its variants [16,17,18,19,20]. Its popularity stems from its simplicity (the model is defined by only five parameters) and its reasonable accuracy. However, one of the main drawbacks of the JA model is its fundamental history-independent (HI) behavior, which results in incorrectly closed minor loops [19,20,21]. This limitation affects the accuracy of the JA model when dealing with distorted excitations.

The most adequate hysteresis models that can address intricate magnetization waveforms are the so-called history-dependent (HD) hysteresis models [1,2,3,4,5,6,7,19]. Many variations of HD hysteresis models exist, where phenomenological hysteresis models are based mostly on the well-known Madelung’s rules, and they are fitted to measured magnetization curves or loops [4,5,6,26]. However, in engineering applications, such models are still not applied widely, mostly because of the complexity of their implementation and/or challenging parameter estimation [4,28,29,30].

A potentially very balanced HD model between complexity and accuracy is the basic HD hysteresis model proposed by Zirka et al. [4]. This formulation of the Zirka–Moroz (ZM) HD hysteresis model allows the model to calculate HD magnetization trajectories within a major hysteresis loop of arbitrary shape, where the incorporation of Madelung’s rules is considered for the behavior of static magnetization curves. Examples of recent works that used the model are [31,32,33,34]. The discussed HD hysteresis model is also implemented into an electromagnetic transient program because of its accuracy and relatively low implementation complexity [30]. A set of recommended parameters that should describe the magnetization curves of a wide range of non-oriented (NO) electrical steel (ES) with acceptable accuracy is recommended in [4]. If the use of this universal parameter set is acceptable, the application of the ZM HD hysteresis model is straightforward. In this case, the initialization of the model requires only the information about the static major loop of specific NO steel sheets [4]. However, if the proposed parameters are not acceptable, additional estimation of parameters must be performed. Fine-tuning the parameters of the ZM HD hysteresis model can be challenging. A set of measured first-order reversal curves (FORCs) is required [30] for the fundamental estimation. The measurement of FORCs is, however, not standardized, and most model users have no access to the dedicated equipment required to perform the measurements.

This research aimed to analyze the fundamental two-step parameter estimation method of the discussed HD hysteresis model systematically. In the first step, we fitted the central model equation to a family of measured FORCs. Based on the results, we fitted the so-called shape variation functions by estimating a set of parameters in the second step. Despite its fundamental nature, this approach had notably not been analyzed previously in the literature. The performed analysis included two different parameter sets: a basic set, consisting of six parameters, and an extended one, consisting of nine parameters. The analysis presents the central mechanism of the presented HD model, the requirements and challenges regarding parameter estimation, and the accuracy and limitations of the discussed HD model systematically. The results include two different estimated parameter sets for two NO and one grain-oriented (GO) ES, which are applied widely in contemporary electric drives and devices. The obtained parameter sets were evaluated and validated with measurements of FORCs, symmetric hysteresis loops, and offset hysteresis loops and compared to the results obtained with the recommended parameter set for NO ESs, as proposed in [4,30]. The main findings highlight the accuracy and challenges of the analyzed two-step estimation approach. The estimated parameter sets were especially accurate for prediction of offset minor loops (OMLs), where the accuracy was also adequate for symmetric minor loops (SMLs). Additionally, the analysis revealed that all of the analyzed ESs were modeled best by considering six parameters, where the additional three parameters did not increase the HD hysteresis model’s accuracy. Lastly, the results confirmed the increased difficulty and limitations encountered when modeling GO ESs, and we assessed the universality of the reference parameter set proposed for the modeling of NO ESs [4,30].

This paper is divided into six sections. The theoretical principles of the discussed HD hysteresis model are given in Section 2. Section 3 contains a description of the analyzed two-step parameter estimation method. The results obtained are presented in Section 4, and discussed in Section 5. Section 6 contains the concluding remarks.

## 2. Theoretical Background

The basic ZM static HD hysteresis model is a phenomenological model. It focuses on capturing and describing the interaction between macroscopic variables, i.e., magnetic flux density *B* and magnetic field strength *H*, based on observed (measured) data rather than deriving these interactions from first principles or theoretical foundations. The primary focus of the model is to consider the well-known Madelung’s rules, which are presented in an expanded fashion in [35,36]. According to these rules, all static magnetization trajectories at the macroscopic level are monotonic, and they have well-defined starting and ending points in the BH-plane, which correspond to the so-called reversal points (RPs). RPs are, thus, fundamental for the implementation of HD magnetization, where the static trajectory according to which magnetization is currently evolving is depending not only on the excitation direction but also on distinct previous magnetization trajectories. Therefore, the modeling of HD magnetization requires the adequate management of storing/deleting information regarding past magnetization, where RPs represent the most basic HD information [5,30,35]. It is important to note, that the so-called static hysteresis models do not consider the rate of change of the excitation waveform, but they are impacted only by the RPs of the excitation waveform. Considering the so-called inverse formulation of magnetization phenomena, where *B* is the independent (input) variable, an arbitrary RP of order *n* is characterized by a point $\left(B_{\mathrm{RP}n},H_{\mathrm{RP}n}\right)$ in the BH-plane.

### 2.1. Branching off from the Previous Magnetization Trajectory

**Figure 1:**
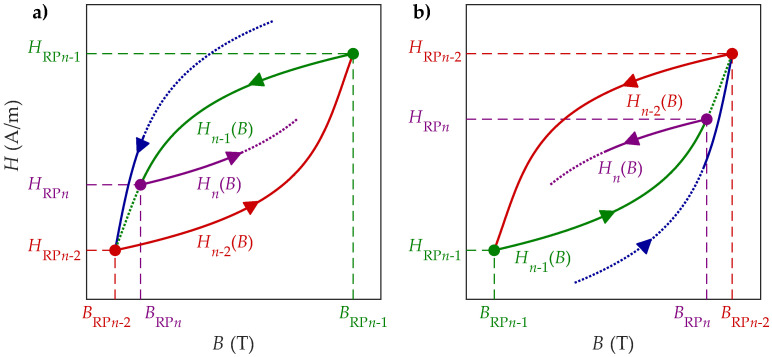
Schematic presentation of branching: (**a**) RC $H_{n}(B)$ is ascending, and (**b**) RC $H_{n}(B)$ is descending.

RPs occur when the magnetization direction is reversed from an increasing to decreasing trend, or vice versa, as the magnetization evolves with time, branching a new magnetization trajectory distinctively in the opposite direction. The RP at $B_{\mathrm{RP}n}$ is the starting point of the reversal curve (RC) $H_{n}(B)$ (also called the *n*-th order RC). This RC starts on the previous RC $H_{n-1}(B)$, where the starting point $H_{n}\left(B_{\mathrm{RP}n}\right)$ can also be denoted as a tip point of a potential hysteresis loop. Thus, the RP at $B_{\mathrm{RP}n}$ ends the evolved part of the RC $H_{n-1}(B)$, where $H_{\mathrm{RP}n}=H_{n}\left(B_{\mathrm{RP}n}\right)=H_{n-1}\left(B_{\mathrm{RP}n}\right)$. The discussed mechanism of the branching with RPs and the corresponding RCs is presented in Figure 1.

It is important to note that RC $H_{n}(B)$ is started only if the reversal occurs before the magnetization corresponding to RC $H_{n-1}(B)$ evolves to its terminal point, in which RC $H_{n-1}(B)$ would close a hysteresis loop. This implies that the starting RP for $H_{n}(B)$ is located between the last two stored RPs. This property holds for both the independent and dependent variables, and for *B*, it is defined by (1):

(1) $$min\left(B_{\mathrm{RP}n-1},B_{\mathrm{RP}n-2}\right)<B_{\mathrm{RP}n}<max\left(B_{\mathrm{RP}n-1},B_{\mathrm{RP}n-2}\right)$$

where both scenarios are considered:

1. $B_{\mathrm{RP}n-1}>B_{\mathrm{RP}n-2}\Rightarrow B_{\mathrm{RP}n-1}>B_{\mathrm{RP}n}$
  In this case, RC $H_{n-1}(B)$ was descending, where *B* decreased from $B_{\mathrm{RP}n-1}$ to $B_{\mathrm{RP}n}$. After the RP at RPn, *B* is increasing, and magnetization evolves according to RC $H_{n}(B)$, as presented in Figure 1a.
2. $B_{\mathrm{RP}n-1}<B_{\mathrm{RP}n-2}\Rightarrow B_{\mathrm{RP}n-1}<B_{\mathrm{RP}n}$
  In this case, RC $H_{n-1}(B)$ was ascending, where *B* increased from $B_{\mathrm{RP}n-1}$ to $B_{\mathrm{RP}n}$. After the RP at $B_{\mathrm{RP}n}$, *B* is decreasing, and magnetization evolves according to RC $H_{n}(B)$, as presented in Figure 1b.

The RP at $B_{\mathrm{RP}n}$ can occur at infinite positions between the limits defined by $B_{\mathrm{RP}n-1}$ and $B_{\mathrm{RP}n-2}$, where the shape of the corresponding RC $H_{n}(B)$ depends on the position of the RP within the discussed limits and, inherently, on all the RPs and RCs of lower orders. Thus, branching is connected directly to the memory-like properties of (soft) magnetic materials, where new RPs and RCs must be stored (added) to the memory stack of the HD model (storing of magnetization history) [5,26,30]. It is important to highlight that the interval on which RCs are defined is decreasing with the increasing order *n* of RPs and RCs (i.e., $\left|B_{\mathrm{RP}n}-B_{\mathrm{RP}n-1}\right|<\left|B_{\mathrm{RP}n-1}-B_{\mathrm{RP}n-2}\right|$). Furthermore, the discussed properties imply that all possible RCs $H_{n}(B)$ are always located within the domain enveloped by RCs $H_{n-1}(B)$ and $H_{n-2}(B)$. Therefore, RCs $H_{n-1}(B)$ and $H_{n-2}(B)$ define the so-called outer loop for $H_{n}(B)$.

### 2.2. Merging of Consecutive Trajectories into a Hysteresis Loop

The arbitrary RC $H_{n}(B)$ ultimately evolves to its terminal point if no RP occurs sooner, i.e., the magnetization evolves until the end of the interval on which the RC is defined. According to Madelung’s rules, the terminal point of RC $H_{n}(B)$ equals the starting point of RC $H_{n-1}(B)$ (that is, *B* evolves to $B_{\mathrm{RP}n-1}$, where $H_{n}\left(B_{\mathrm{RP}n-1}\right)=H_{n-1}\left(B_{\mathrm{RP}n-1}\right)$). This property is generally known as *the return point rule* [7,35,36]. The path of RC $H_{n}(B)$ is different from that of RC $H_{n-1}(B)$, forming two monotonic and non-intersecting curves. Therefore, these RCs form a properly closed hysteresis loop that spans between the RPs $\left(B_{\mathrm{RP}n-1},H_{\mathrm{RP}n-1}\right)$ and $\left(B_{\mathrm{RP}n},H_{\mathrm{RP}n}\right)$.

As soon as a hysteresis loop is properly closed, the evolution of magnetization follows the RC $H_{n-2}(B)$ as if the closed hysteresis loop never existed (i.e., the RC $H_{n-1}(B)$ and $H_{n}(B)$ never existed), continuing from $B_{\mathrm{RP}n-1}$. Because the starting point of $H_{n-2}(B)$ is already defined by $B_{\mathrm{RP}n-2}$ within the memory, RPs and RCs corresponding to $B_{\mathrm{RP}n-1}$ and $B_{\mathrm{RP}n}$ are irrelevant for the future evolution of magnetization and can be deleted. This is generally known as *the wiping-out property* [7,35,36]. Thus, closing a hysteresis loop properly is also connected directly to the memory-like properties of (soft) magnetic materials, where the last two RPs and RCs must be deleted from the memory stack of the HD model (deleting of magnetization history) [5,26,30]. It is worth highlighting that the interval on which RCs are defined is increased when a hysteresis loop is properly closed.

### 2.3. History-Independent Magnetization

The evolution of magnetization along an arbitrary RC is, in general, always disrupted either by branching (an RP occurs before the respective RC evolves until its terminal point and magnetization evolves according to a new RC) or by merging (the respective RC evolves until its terminal point, and magnetization continues along the antepenultimate RC in respect to the respective RC that closed the hysteresis loop), as discussed in Section 2.1 and Section 2.2. Based on these two mechanisms, HD magnetization can be modeled only if adequate initial conditions are known.

A special case of magnetization occurs if the magnetic material is saturated. In the fully saturated state, magnetization evolves according to a simple anhysteretic trajectory, generally defined by a single-valued function $H_{\mathrm{sat}}(B)$ for positive saturation and $-H_{\mathrm{sat}}(-B)$ for negative saturation. Therefore, the evolution of magnetization in saturation is HI [4,5,35]. Saturation can only be reached by increasing excitation excessively in the positive or the negative direction (i.e., $B\geq B_{\mathrm{sat}}$ or $B\leq -B_{\mathrm{sat}}$). Such an increase implies that *B* is extended beyond all the stored RPs, where, in the evolution of magnetization, all the hysteresis loops corresponding to the stored RPs are closed, and all magnetization history is deleted by merging, as discussed in Section 2.2.

Consequently, the evolution of magnetization always follows the same trajectory when starting from saturation. If *B* is evolving from $-B_{\mathrm{sat}}$ towards $B_{\mathrm{sat}}$, magnetization evolves according to the ascending branch $H_{a}\left(B\right)$ of the so-called major hysteresis loop. In the opposite direction, i.e., if *B* is evolving from $B_{\mathrm{sat}}$ towards $-B_{\mathrm{sat}}$, magnetization evolves according to the descending branch $H_{d}\left(B\right)$ of the major loop. When cyclic magnetization between negative and positive saturation is generated, both branches form the major hysteresis loop, which is the biggest possible hysteresis loop. It spans between the fully saturated states of the material, where the starting RPs of $H_{a}\left(B\right)$ and $H_{d}\left(B\right)$ correspond to $-B_{\mathrm{sat}}$ and $B_{\mathrm{sat}}$, respectively. The major loop is symmetric (i.e., $H_{a}\left(B\right)=-H_{d}\left(-B\right)$), where both the ascending branch and the descending branch are HI. Therefore, these RCs are fundamental for the initialization of HD magnetization, where the odd symmetry can be exploited to determine the HD magnetization curves of the first order.

All RCs that start on one of the major loop branches are generally denoted as first-order RCs (FORCs) and represent the fundamental HD RCs $H_{1}\left(B\right)$, where n=1. According to the return point rule, FORCs have corresponding terminal points in saturation, i.e., at $-B_{\mathrm{sat}}$ or $B_{\mathrm{sat}}$ [4]. Further, as explained in Section 2.1, if an RP occurs on a FORC, the magnetization evolves corresponding to a second-order RC (SORC) $H_{2}\left(B\right)$, where n=2. If a hysteresis loop is closed with an SORC, all magnetization history is also deleted, where magnetization evolves further along the major loop. The further evolution of HD magnetization is governed by the branching and merging mechanisms presented in Section 2.1 and Section 2.2.

### 2.4. Modeling of HD Magnetization Trajectories

The basic assumption of the presented ZM HD hysteresis model is that the measured major hysteresis loop data are available and they are modeled by functions describing the ascending $H_{a}\left(B\right)$ and descending $H_{d}\left(B\right)$ branches. The ZM hysteresis model focuses on the description of HD magnetization curves inside the major loop [4].

**Figure 2:**
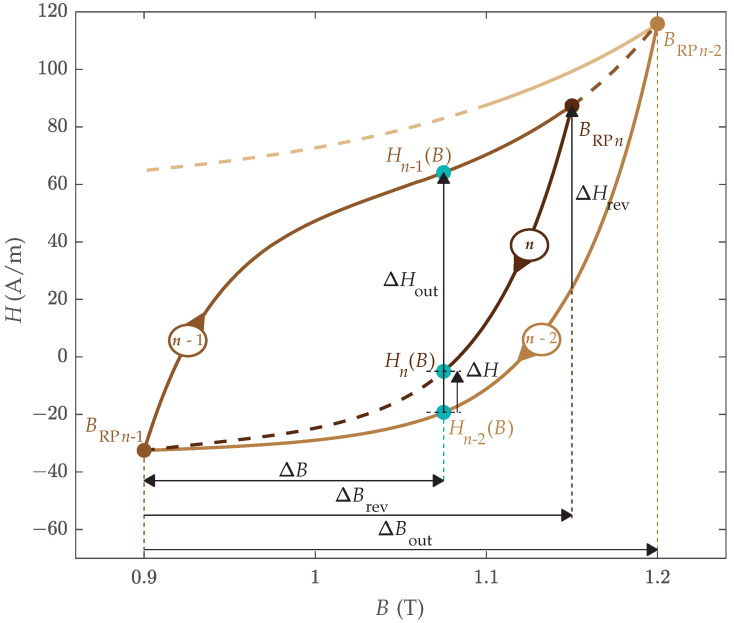
Schematic representation of the RC under construction $H_{n}\left(B\right)$, highlighting all the variables necessary to determine $H_{n}\left(B\right)$.

The basic element for the construction of the HD magnetization curve $H_{n}(B)$ is the so-called outer loop, which is composed of the last two magnetization curves $H_{n-1}(B)$ and $H_{n-2}(B)$ that preceded the magnetization curve under construction, as discussed in Section 2.1. $H_{n-1}(B)$ and $H_{n-2}(B)$ both span between $B_{\mathrm{RP}n-2}$ and $B_{\mathrm{RP}n-1}$ and envelope $H_{n}(B)$, as presented in Figure 2. Therefore, relevant previous RPs and RCs must be stored in the memory stack of the HD hysteresis model according to the discussed branching and merging rules [30].

The magnetization curve under construction $H_{n}(B)$ is defined from $B_{\mathrm{RP}n}$ to $B_{\mathrm{RP}n-1}$, where the position of $B_{\mathrm{RP}n}$ can be arbitrary between $B_{\mathrm{RP}n-1}$ and $B_{\mathrm{RP}n-2}$. The arbitrary length of the discussed interval necessitates introducing a normalized independent variable *b*, which enables the implementation of generalized mathematical functions for the modeling of different RCs (in the original model description in [4], this variable was denoted by *x*. However, introducing a more intuitive convention, in this paper, the discussed variable is denoted by *b*, since it represents the normalized independent variable *B*). The normalized independent variable *b* is defined based on min–max normalization by (2)

(2) $$b=\frac{\Delta B}{\Delta B_{\mathrm{rev}}}=\frac{B_{\mathrm{RP}n-1}-B}{B_{\mathrm{RP}n-1}-B_{\mathrm{RP}n}}$$

and decreases from 1 at $B=B_{\mathrm{RP}n}$ to 0 at $B=B_{\mathrm{RP}n-1}$, regardless of the order *n*, size of the interval $\left[B_{\mathrm{RP}n},B_{\mathrm{RP}n-1}\right]$, and position of $B_{\mathrm{RP}n}$.

The shape of the curve under construction is modeled further considering the width of the corresponding outer loop $\Delta H_{\mathrm{out}}\left(B\right)$. This approach considers all shapes of previous magnetization curves implicitly when modeling $H_{n}(B)$, beginning from the major hysteresis loop. The width of the outer loop $\Delta H_{\mathrm{out}}\left(B\right)$ is defined by (3):

(3) $$\Delta H_{\mathrm{out}}\left(B\right)=H_{n-2}\left(B\right)-H_{n-1}\left(B\right)$$

where $\Delta H_{\mathrm{out}}\left(B\right)$ varies from $\Delta H_{\mathrm{out}}\left(B_{\mathrm{RP}n}\right)=\Delta H_{\mathrm{rev}}$ to $\Delta H_{\mathrm{out}}\left(B_{\mathrm{RP}n-1}\right)=0$.

Further, the magnetization curve under construction $H_{n}(B)$ is, with the evolution of magnetization, approaching RC $H_{n-2}(B)$, where the terminal point at $B_{\mathrm{RP}n-1}$ is located on RC $H_{n-2}(B)$, i.e., $H_{n}\left(B_{\mathrm{RP}n-1}\right)=H_{n-1}\left(B_{\mathrm{RP}n-1}\right)=H_{n-2}\left(B_{\mathrm{RP}n-1}\right)$. The shapes of RCs $H_{n}(B)$ and $H_{n-2}(B)$ are similar, i.e., both RCs are either ascending or descending, as presented in Figure 1 and Figure 2. Therefore, the curve under construction is modeled based on the difference ΔH between these curves, as defined by (4).

(4) $$H_{n}\left(B\right)=H_{n-2}\left(B\right)-\Delta H\left(B,B_{\mathrm{RP}n},B_{\mathrm{RP}n-1}\right)$$

The difference $\Delta H\left(B,B_{\mathrm{RP}n},B_{\mathrm{RP}n-1}\right)$ is always approaching zero with the evolution of *b*; therefore, it can be modeled by relatively simple mathematical functions. The authors of [4] proposed to model the decreasing HD difference $\Delta H\left(B,B_{\mathrm{RP}n},B_{\mathrm{RP}n-1}\right)$ by (5):

(5) $$\Delta H\left(B,B_{\mathrm{RP}n},B_{\mathrm{RP}n-1}\right)=\Delta H_{\mathrm{out}}\left(B_{\mathrm{RP}n}\right)\left(1-p_{2}\right)b^{-p_{1}\left(1-b\right)}+\Delta H_{\mathrm{out}}\left(B\right)p_{2}b^{p_{3}}$$

where *B* is evolving from $B_{\mathrm{RP}n}$ towards $B_{\mathrm{RP}n-1}$; *b* is evolving according to (2); $p_{1}$, $p_{2}$, and $p_{3}$ are determining the shape of the curve under construction (in the original model description in [4], these variables were denoted by *a*, *b*, and *c*, where in this paper, $p_{1}\equiv a$, $p_{2}\equiv b$, and $p_{3}\equiv c$). By determining $H_{n}(B)$ based on $H_{n-2}(B)$ and considering RPs $B_{\mathrm{RP}n},B_{\mathrm{RP}n-1}$, the shapes of the past magnetization impact the shape of the RC under construction. Physical behavior is ensured by limiting $p_{1}>0$, $0<p_{2}<1$, and $p_{3}>0$ [4].

### 2.5. Considering the Position-Based Shape Variations of Reversal Curves

The HD model based on (5) can model individual HD RCs with high accuracy by estimating $p_{1}$, $p_{2}$, and $p_{3}$ adequately. However, a fixed set of values does not guarantee high accuracy for all the possible RCs (i.e., the family of RCs) within the outer loop. The values of $p_{1}$, $p_{2}$, and $p_{3}$ depend on the position of the RC within the outer loop (i.e., they depend on the position of the ultimate RP). For this reason, they must be expressed as adequate shape variation functions of variables that represent the position of the RC within the outer loop. The authors applied two variables, which correspond to the position of $H_{n}(B)$ within the outer loop [4]. The first is the width of the interval on which $H_{n}\left(B\right)$ is constructed, i.e., $\Delta B_{\mathrm{rev}}$, which represents the distance between the ultimate and penultimate RP, and it is defined in (2). The second variable is the relative position of RPs β, as defined by (6):

(6) $$\beta =\frac{\Delta B_{\mathrm{rev}}}{\Delta B_{\mathrm{out}}}=\frac{B_{\mathrm{RP}n-1}-B_{\mathrm{RP}n}}{B_{\mathrm{RP}n-1}-B_{\mathrm{RP}n-2}}$$

where $\Delta B_{\mathrm{out}}$ is the height of the corresponding outer loop.

Based on $\Delta B_{\mathrm{rev}}$ and β, the authors in [30] proposed to model the position-based shape variations in RCs by introducing shape variation functions (7)–(9).

(7) $$p_{1}\left(\Delta B_{\mathrm{rev}},\beta \right)=\left|\Delta B_{\mathrm{rev}}\right|\left(y_{1}+y_{2}\beta +y_{3}\beta^{2}+y_{4}\beta^{3}\right)$$

(8) $$p_{2}\left(\beta \right)=y_{5}\left(1-\beta \right)+y_{6}{\left(1-\beta \right)}^{2}+y_{7}{\left(1-\beta \right)}^{3}$$

(9) $$p_{3}\left(\beta \right)=y_{8}+y_{9}\beta$$

The presented description was developed for the general case, which included the modeling of both NO and GO ES, where a set of nine parameters, $y_{9}=\left[y_{1},\ldots ,y_{9}\right]$, was introduced for the modeling of GO ES [30]. Additionally, the authors proposed a simplified description with six parameters, $y_{6}=\left[y_{1},\ldots ,y_{5},y_{8}\right]$, where in (8) and (9), terms including $y_{6}$, $y_{7}$, and $y_{9}$ were neglected. The latter description was intended for NO ESs [4]. For the general purpose, y is used to replace $y_{9}$ or $y_{6}$ hereafter, if applicable.

### 2.6. Initialization of the HD Model, First Magnetization Curve, and Symmetric Minor Loops

The fundamental assumption regarding the initial condition of the discussed model is that the evolution of magnetization starts from a fully saturated, i.e., HI, state [4]. The shape of the major loop, in combination with the RPs of the excitation waveform, determines the corresponding HD magnetization trajectories. Consequently, if magnetization that is starting from a point within the major loop is required, preliminary demagnetization is required from saturation to the discussed point. Such an initial condition is HD, as it can be reached by theoretically infinite demagnetization profiles, where the magnetization from such an initial condition will adhere to the preliminary demagnetization profile [4].

The most often required unsaturated initial conditions correspond to either properly demagnetized material (i.e., the initial condition corresponds to the origin of the BH coordinate system) or to a point on the first magnetization curve. The first magnetization curve is, in general, an HI magnetization trajectory, fundamental for the description of material properties [5]. It is important to note that the discussed ZM HD model does not consider the first magnetization curve as a model input but rather models this curve implicitly. This can be obtained by symmetric cyclic demagnetization with an adequately small demagnetization step until the fully demagnetized state of the material is reached [4]. The first magnetization curve can be obtained starting from this state by unidirectional magnetization to saturation. Furthermore, SMLs can be predicted by cyclic magnetization, starting from the corresponding loop tip at the discussed first magnetization curve.

## 3. Estimation of ZM Model’s Parameters

The goal of parameter estimation is to tune the parameter set y in such a way that the various calculated HD magnetization curves match the measured curves with high accuracy [29,30]. The parameter estimation of phenomenological models is based on measured data, where selected macroscopic static magnetization trajectories can be applied in different parameter estimation approaches [28,29]. Accurate and efficient parameter estimation demands consistency between the available data and the estimation approach.

### 3.1. Estimation of Parameters Based on Symmetric Minor Loops

Standard methods for measuring the magnetic properties of ES sheets are outlined in the International Standards IEC 60404-2, which specifies the use of an Epstein Frame, and IEC 60404-3, which details the methods for Single Sheet Testers. Standardized Epstein Frame and Single Sheet Tester methods offer reproducible measurements that are essential for industrial applications, such as quality control and material classification. These methods are used primarily to measure specific losses and symmetric hysteresis loops, which are fundamental for evaluating commercial ESs. These methods can be applied to measure the so-called quasi-static symmetric loops, which are measured at adequately low excitation frequencies, where the influence of induced eddy currents remains negligible [37]. The symmetric loop with the highest amplitude is generally limited by the excitation capability of the measurement system, and it is often regarded as a good approximation of the major loop. Assuming that a set of symmetric loops including the approximated major loop is available, the estimation can be performed by fitting the model predictions to the measured SMLs, where the deviation between the calculated and measured loops is minimized. In this way, the parameter set y is estimated directly. A similar estimation approach was proposed in [30]. However, the primary drawback of such an estimation approach is the necessity to initialize the model adequately (i.e., perform adequate demagnetization routine) at each optimization step before calculating all minor loops. This requirement can make the estimation process highly computationally intensive and time-consuming. Additionally, SMLs do not necessarily cover the entire range of β, which compromises the fitting of shape variation functions. Thus, parameter estimation must be supplemented with additional extrapolation functions, as demonstrated in [30].

### 3.2. Estimation of Parameters Based on the Measured FORCs

Computational complexity can be reduced significantly by avoiding the calculation of many HD RCs of high orders in the preliminary demagnetization. Instead, estimation can be performed based on the most fundamental HD RCs, specifically FORCs. In comparison to SMLs, FORCs provide more detailed information on the behavior of magnetic domains and their interactions [38,39,40], which can be crucial for the research and development of magnetic materials. However, FORC measurements are more complex and require sophisticated analysis (e.g., analyzing FORC diagrams), making them less suitable for routine industrial testing. Specifically, FORC diagrams are used rarely in the phenomenological modeling of hysteresis, but they are, e.g., useful in estimating the parameters of the Preisach model [39,40]. One of the main challenges is that the quasi-static measurements of FORCs involve low-frequency excitations. At such low frequencies, the combination of small signals (with potentially low signal-to-noise ratios) and long integration times imposes tight control on the drift in the measurement circuit. This drift can accumulate over time, leading to significant errors. Stable electronic components and sensors must be employed to mitigate this. If some residual drift occurs, it must be corrected after analog-to-digital conversion through linear numerical compensation over individual periods [40,41].

The main advantage is that the FORC-based estimation approach simplifies the fundamental HD mechanism described by (2)–(5). In this scenario, the outer loop for constructing all FORCs corresponds to the HI major loop, where the HI ascending branch $H_{a}\left(B\right)$ and the HI descending branch $H_{d}\left(B\right)$ serve as inputs for parameter estimation. The FORCs can be generated starting either from $H_{a}\left(B\right)$ (the corresponding FORCs are descending) or from $H_{d}\left(B\right)$ (the corresponding FORCs are ascending), where analogous descending and ascending FORCs are symmetric in respect to the origin of the BH plane. Therefore, the odd symmetry allows measurements of the FORCs in an arbitrary direction [30].

Considering that measured FORCs are ascending and that the major loop spans between $\pm B_{\mathrm{sat}}$, an arbitrary FORC starts at $B_{\mathrm{RP}1}$, where $-B_{\mathrm{sat}}<B_{\mathrm{RP}1}<B_{\mathrm{sat}}$, and ends at $B_{\mathrm{sat}}$. Consequently, Equations (2)–(6) are simplified into to (10)–(13). The normalized input variable *b* changes from 1 (i.e., $B=B_{\mathrm{RP}1}$) to 0 (i.e., $B=B_{\mathrm{sat}}$) and is defined by (10):

(10) $$b=\frac{\Delta B}{\Delta B_{\mathrm{rev}}}=\frac{B_{\mathrm{sat}}-B}{B_{\mathrm{sat}}-B_{\mathrm{RP}1}}$$

where $\Delta B_{\mathrm{rev}}>0$, and corresponds to the length of the interval $\left[B_{\mathrm{RP}1},B_{\mathrm{sat}}\right]$ on which the observed FORC is defined.

The outer loop width function $\Delta H_{\mathrm{out}}\left(B\right)$ corresponds to the width of the major loop, and is defined by (11).

(11) $$\Delta H_{\mathrm{out}}\left(B\right)=H_{a}\left(B\right)-H_{d}\left(B\right)$$

By combining(4) and (5), the modeled FORC $H_{1}\left(B\right)$ is finally defined by (12).

(12) $$H_{1}\left(B\right)=H_{a}\left(B\right)-\left(\Delta H_{\mathrm{out}}\left(B_{\mathrm{RP}1}\right)\left(1-p_{2}\right)b^{-p_{1}\left(1-b\right)}+\Delta H_{\mathrm{out}}\left(B\right)p_{2}b^{p_{3}}\right)$$

According to (10)–(12), individual FORCs can be calculated based directly on $H_{a}\left(B\right)$ and $H_{d}\left(B\right)$; RP $B_{\mathrm{RP}1}$; and adequate values of $p_{1}$, $p_{2}$, and $p_{3}$. Therefore, $p_{1}$, $p_{2}$, and $p_{3}$ can be estimated by fitting (10) through (12) to the corresponding measured FORC.

**Figure 3:**
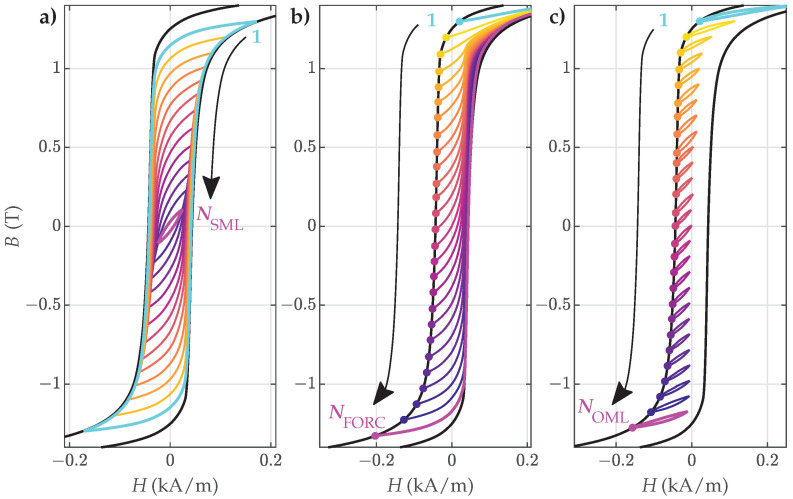
Schematic presentation of the measured curves (based on the measurements for the NO32 ES): (**a**) SMLs color-coded and enumerated from 1 to $N_{\mathrm{SML}}$; (**b**) FORCs color-coded and enumerated from 1 to $N_{\mathrm{FORC}}$, according to the increasing β; (**c**) OMLs color-coded and enumerated from 1 to $N_{\mathrm{OML}}$. The major loop is presented as a solid black line.

Theoretically, infinite FORCs exist within the major loop, with significantly different shapes. Individual FORCs within the family can be classified further by $\Delta B_{\mathrm{rev}}$, and by the ratio β, they are defined by (13):

(13) $$\beta =\frac{\Delta B_{\mathrm{rev}}}{\Delta B_{\mathrm{out}}}=\frac{B_{\mathrm{sat}}-B_{\mathrm{RP}1}}{B_{\mathrm{sat}}-\left(-B_{\mathrm{sat}}\right)}=\frac{B_{\mathrm{sat}}-B_{\mathrm{RP}1}}{2B_{\mathrm{sat}}}$$

where $\Delta B_{\mathrm{out}}=2B_{\mathrm{sat}}$ is equal for all FORCs. An exemplary family of measured FORCs is presented in Figure 3b.

### 3.3. A Two-Step Estimation Approach

The proposed approach gives increased insight into the fitting capability and limitations of the main model (Equation (5)), which is fitted in the first step to individual members of the measured family of FORCs. In the second step, the shape variation functions $p_{1}\left(\Delta B_{\mathrm{rev}},\beta \right)$, $p_{2}\left(\beta \right)$, and $p_{3}\left(\beta \right)$, defined by (7) to (9), are fitted to the results obtained in the first step. The results obtained in the first step inform the selection of fitting functions for considering shape variations versus β, where different sets of y can be applied, e.g., the full set with nine or a reduced set with six parameters, as discussed in Section 2.5.

The FORC model defined by (10)–(12) can be fitted to adequate measured data. Each measured FORC is characterized by its RP $B_{\mathrm{RP}1}$, and it is measured in $N_{p}$ measurement points between $B_{\mathrm{RP}1}$ and $B_{\mathrm{sat}}$. The obtained results can be arranged in a vector containing all the values of the independent variable $D_{\mathrm{Bmeas}}=\left[B_{\mathrm{meas},1},\ldots ,B_{\mathrm{meas},N_{p}}\right]$ at which the material was evaluated and a vector of corresponding values of the dependent variable $D_{\mathrm{Hmeas}}=\left[H_{\mathrm{meas},1},\ldots ,H_{\mathrm{meas},N_{p}}\right]$. The values of $p_{1}$, $p_{2}$, and $p_{3}$ corresponding to the measured FORC can finally be obtained by minimizing the cost function $F_{\mathrm{step}1}$, as defined by (14).

(14) $$F_{\mathrm{step}1}=\sum_{i=1}^{N_{p}}{\left[H_{\mathrm{meas},i}-H_{1}\left(B_{\mathrm{meas},i},p_{1},p_{2},p_{3}\right)\right]}^{2}$$

In the first step, the estimation of $p_{1}$, $p_{2}$, and $p_{3}$ is performed based on several measured FORCs corresponding to various β (that is, RPs at different values of $B_{\mathrm{RP}1}$). The obtained values are grouped into three vectors, i.e., $D_{p1}=\left[p_{1,1},\ldots ,p_{1,N_{\mathrm{RC}}}\right]$, $D_{p2}=\left[p_{2,1},\ldots ,p_{2,N_{\mathrm{RC}}}\right]$ and $D_{p3}=\left[p_{3,1},\ldots ,p_{3,N_{\mathrm{RC}}}\right]$, where $N_{\mathrm{RC}}$ is the number of measured FORCs. For each FORC, the corresponding $\Delta B_{\mathrm{rev}}$ and β are calculated using (13), and the data for all considered FORCs are arranged into the vectors $D_{\Delta \mathrm{Brev}}=\left[\Delta B_{\mathrm{rev},1},\ldots ,\Delta B_{\mathrm{rev},N_{\mathrm{RC}}}\right]$ and $D_{\beta }=\left[\beta_{1},\ldots ,\beta_{N_{\mathrm{RC}}}\right]$. All data are arranged in increasing order in respect to β. The obtained vectors are the basis for the second step, i.e., the estimation of the parameter set $y_{6}$ or $y_{9}$. The estimation in the second step is based on fitting (7)–(9) individually by minimizing the cost function $F_{\mathrm{step}2}$, as defined by (15):

(15) $$F_{\mathrm{step}2}=\sum_{i=1}^{N_{\mathrm{RC}}}{\left[p_{•,i}-p_{•}\left(\Delta B_{\mathrm{rev},i},\beta_{i},y\right)\right]}^{2}$$

where • is a placeholder for denoting either $p_{1}$, $p_{2}$, or $p_{3}$.

## 4. Results

We analyzed the proposed estimation approach by examining three types of ESs with varying properties: NO ES sheets with thicknesses of 0.27 mm (denoted by NO27 ES) and 0.32 mm (denoted by NO32 ES) and GO ES sheets with a thickness of 0.27 mm (denoted by GO27 ES). Such ES sheets are used predominantly in contemporary high-performance electric drives and electromagnetic devices. All ES samples were evaluated experimentally within a Single Sheet Tester setup. The excitation dynamics were adequately low to ensure quasi-static magnetization conditions, where the fundamental excitation frequency was lower than 0.05 Hz. In order to perform a systematic analysis, we measured the following families of magnetization trajectories:

(a) Symmetric hysteresis loops, presented schematically in Figure 3a: The biggest symmetric loop for individual material samples was assumed as the major loop (i.e., the loop tips were assumed $\pm B_{\mathrm{sat}}$) and was used as the model input. The remaining SMLs were used for the validation of the estimated parameters.
(b) First-order reversal curves (FORCs), presented schematically in Figure 3b: A family of FORCs was measured within the assumed major loop. These represented the basis for the proposed two-step estimation of the parameters.
(c) Offset minor loops (OMLs) along the assumed major loop, presented schematically in Figure 3c: These were used for the validation of the estimated parameters.

All measured curves were obtained based on the equidistant distribution of $B_{\mathrm{RP}n}$ between $\pm B_{\mathrm{sat}}$. Table 1 provides an overview of the measured RCs. In this context, $B_{\mathrm{sat}}$ represented the highest measured magnetic flux density of the largest symmetric hysteresis loop, which was considered the major loop. $H_{\mathrm{sat}}$ denotes the corresponding magnetic field strength, which was constrained by the limitations of the measurement system. The number of measured SMLs, FORCs, and OMLs is denoted by $N_{\mathrm{SML}}$, $N_{\mathrm{FORC}}$, and $N_{\mathrm{OML}}$, respectively.

Finally, the measured data were organized considering individual curves and grouped into dataset vectors $D_{\mathrm{Bmeas}}$ and $D_{\mathrm{Hmeas}}$, as discussed in Section 3.3. To evaluate the goodness of fit of all different curves, we applied the normalized root mean square (NRMS) statistical measure ε, as defined by (16) [20]:

(16) $$\varepsilon =\sqrt{\frac{1}{N_{\max}}\sum_{i=1}^{N_{\max}}{\left(\frac{e_{H,i}}{\Delta H_{\mathrm{eval}}}\right)}^{2}}=\sqrt{\frac{1}{N_{\max}}\sum_{i=1}^{N_{\max}}{\left(\frac{H_{\mathrm{meas},i}-H_{\mathrm{calc},i}}{\Delta H_{\mathrm{eval}}}\right)}^{2}}$$

where $N_{\max}$ is the number of evaluation points along the analyzed RC. Furthermore, $e_{H,i}=H_{\mathrm{meas},i}-H_{\mathrm{calc},i}$ is the difference between the measured and predicted values (i.e., the residual) in individual evaluation points, where $H_{\mathrm{meas},i}$ corresponds to the measured value of the magnetic field strength, and $H_{\mathrm{calc},i}$ is the corresponding calculated value. Finally, $\Delta H_{\mathrm{eval}}$ is the difference between the maximum and minimum values (i.e., the difference between the starting and end points) of the evaluated RC.

### 4.1. First Step of the Estimation Approach

In the first step, the values of $p_{1}$, $p_{2}$, and $p_{3}$ were calculated based on all the measured FORCs for the three discussed ES samples. A dataset was obtained by implementing the cost function (14) and the model (12) within the lsqnonlin function in Matlab 2024b and solving the optimization problem for individual FORCs. The obtained datasets were organized into vectors $D_{p1}$, $D_{p2}$, $D_{p3}$, $D_{\Delta \mathrm{Brev}}$, and $D_{\beta }$, as discussed in Section 3.3.

**Figure 4:**
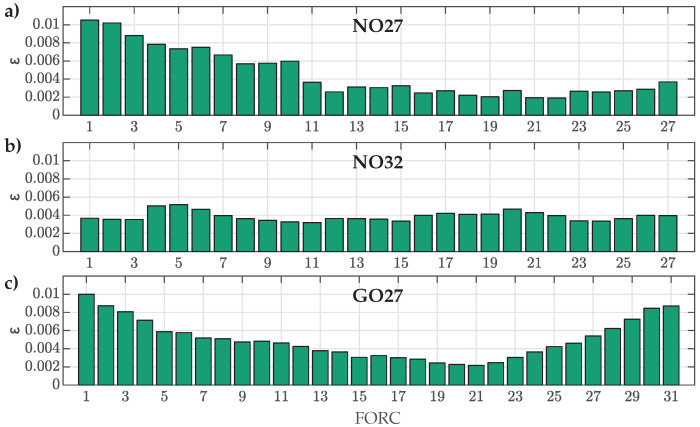
Comparison of calculated NRMS deviation ε between individual measured and corresponding fitted FORCs in the first estimation step for (**a**) NO27 ES, (**b**) NO32 ES, and (**c**) GO27 ES.

The obtained data were first used to analyze the ability of the main model function (12) to fit the various measured FORCs for all three ES samples. For this purpose, NRMS deviations between all measured curves and corresponding optimal fits were calculated by (16). The results are presented in Figure 4.

The calculated NRMS deviations ε confirmed that the main model function (12) is generally adequate for the overall description of various FORCs for all three analyzed ES samples. The goodness of fit was, in general, better for FORCs with RPs in the middle of the major loop, especially in the case of the GO27 ES. Furthermore, the poorest overall goodness of fit was observed in the case of NO27 ES, where ε was more than twice as high for FORC Nos. 1–6 compared to NO32 ES.

**Figure 5:**
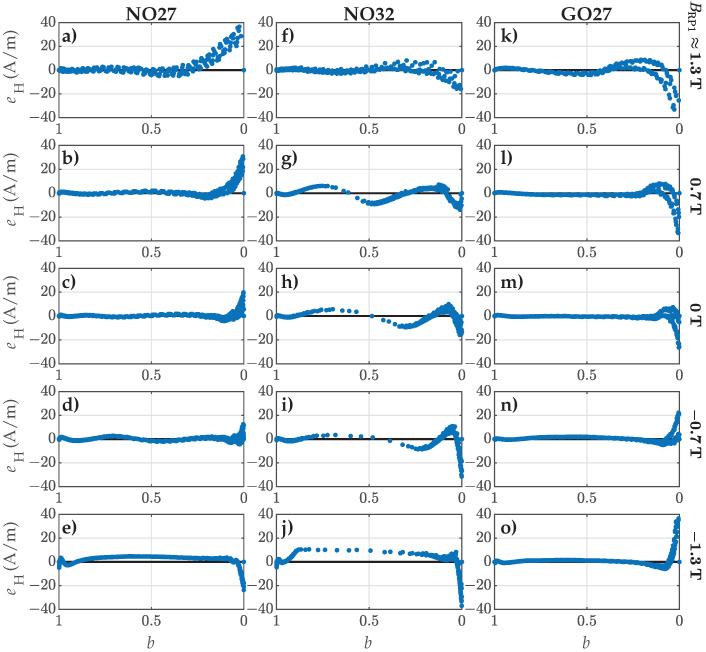
Comparison of the calculated residuals $e_{H}$ along selected FORCs. The rows correspond to comparable values of $B_{\mathrm{RP}1}$, and the columns correspond to individual ES samples: (**a**–**e**) NO27 ES (FORC No. 1, 7, 14, 21, and 27), (**f**–**j**) NO32 ES (FORC No. 1, 7, 14, 21, and 27), and (**k**–**o**) GO27 ES (FORC No. 3, 9, 16, 23, and 29).

Additionally, we analyzed the residuals $e_{H}$ along the FORCs in respect to *b*. Figure 5 presents the results for the five selected FORCs. Subplots (a–e) correspond to FORC Nos. 1, 7, 14, 21, and 27 for NO27 ES. Analogously, the subplots (f–j) correspond to FORC Nos. 1, 7, 14, 21, and 27 for NO32 ES, while the subplots (k–o) depict FORC Nos. 3, 9, 16, 23, and 29 for GO27 ES. The residuals are presented systematically for decreasing values of *b*, starting at b=1 (representing the starting points of FORCs at $B_{\mathrm{RP}1}$) and ending at b=0 (corresponding to the end points of FORCs at $B_{\mathrm{sat}}$). Furthermore, the individual rows in Figure 5 correspond to FORCs with comparable values of $B_{\mathrm{RP}1}$, whereas the individual columns correspond to the three analyzed materials.

The presented results provided further insight on the fitting accuracy beyond ε for individual FORCs and individual ES samples. The obtained results demonstrated clearly that the model was unable to fit the measured curves perfectly. The most significant deviations were observed consistently at flux densities nearing saturation (i.e., the so-called knee region and beyond, where b→0), where the magnetization curves exhibited the highest degree of nonlinearity. The limitations of the model had a significant impact on the obtained fits. In the case of the NO27 ES, deviations in the knee region led to an increase in ε, particularly for FORC Nos. 1 to 10. Similarly, an increase in ε was observed for GO27 ES with FORC Nos. 25 to 31, additionally exhibiting a steady rise in ε. Although the NRMS deviation for NO32 ES was comparable across all the analyzed FORCs, the results in Figure 4 revealed a significant mismatch in the slopes of the modeled FORCs. This discrepancy was evident as the measured points displayed oscillating trends around the corresponding modeled trajectories with respect to *b*.

For the FORCs with the highest $\Delta B_{\mathrm{rev}}$ (i.e., FORC Nos. 27, 27, and 31), the residuals indicated that the modeled curves were substantially offset in *H* relative to the measured ones, particularly for both the analyzed NO ES samples. These FORCs exhibited the highest overall nonlinearity, resulting in the poorest fits. It is important to note that this conclusion cannot be drawn from the analysis based solely on ε.

### 4.2. Second Step of the Estimation Approach

In the second step, datasets $D_{p1}$, $D_{p2}$, $D_{p3}$, $D_{\Delta \mathrm{Brev}}$, and $D_{\beta }$ were used for the estimation of y, as discussed in Section 3.3. Both the extended parameter set $y_{9}$ and the reduced parameter set $y_{6}$ were estimated for all three ES samples by implementing the cost function (15) and the functions (7)–(9) within the lsqnonlin function in Matlab 2024b. The calculated model parameter sets are gathered in Table 2.

The results gathered in Table 2 were further compared to the reference parameter values $y_{6,\mathrm{ref}}$ in [30], which are, according to the authors of the model, adequate for the modeling of a variety of NO ES samples. The comparison revealed a notable deviation from the parameter sets estimated using the presented two-step approach. Nevertheless, all estimated parameters of $y_{6}$ remained comparable in terms of their order of magnitude.

**Figure 6:**
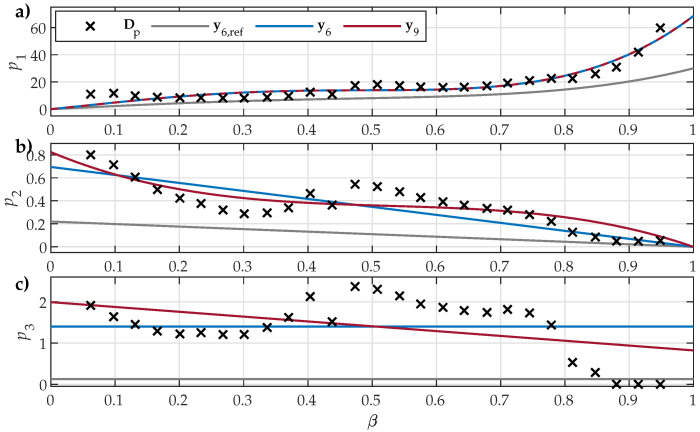
Comparison of the fitted shape variation functions for NO27 ES when considering $y_{6,\mathrm{ref}}$, $y_{6}$, or $y_{9}$: (**a**) $p_{1}\left(\beta \right)$ compared to $D_{p1}\left(\beta \right)$; (**b**) $p_{2}\left(\beta \right)$ compared to $D_{p2}\left(\beta \right)$; (**c**) $p_{3}\left(\beta \right)$ compared to $D_{p3}\left(\beta \right)$. The numeric values of $D_{p1}$, $D_{p2}$, and $D_{p3}$ were obtained in the first estimation step.

**Figure 7:**
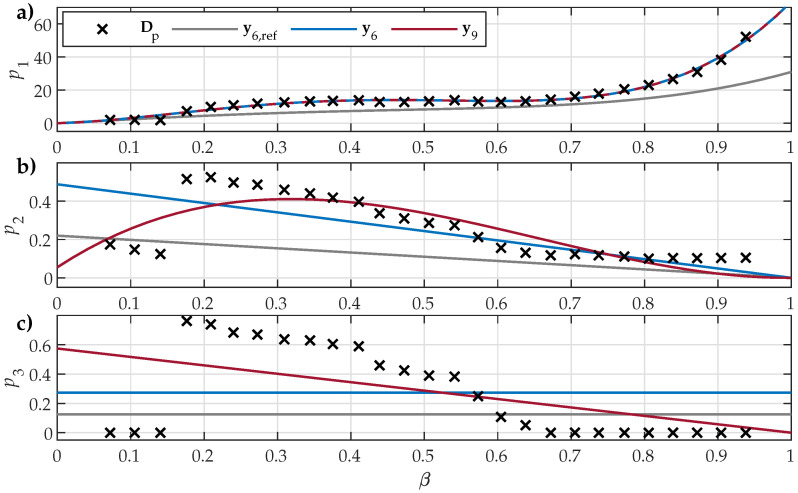
Comparison of the fitted shape variation functions for NO32 ES when considering $y_{6,\mathrm{ref}}$, $y_{6}$, or $y_{9}$: (**a**) $p_{1}\left(\beta \right)$ compared to $D_{p1}\left(\beta \right)$; (**b**) $p_{2}\left(\beta \right)$ compared to $D_{p2}\left(\beta \right)$; and (**c**) $p_{3}\left(\beta \right)$ compared to $D_{p3}\left(\beta \right)$. The numeric values of $D_{p1}$, $D_{p2}$, and $D_{p3}$ were obtained in the first estimation step.

**Figure 8:**
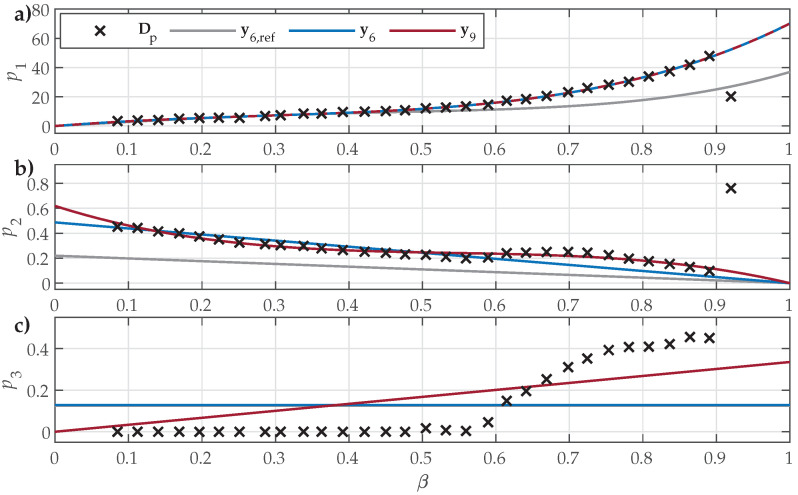
Comparison of the fitted shape variation functions for GO27 ES when considering $y_{6,\mathrm{ref}}$, $y_{6}$, or $y_{9}$: (**a**) $p_{1}\left(\beta \right)$ compared to $D_{p1}\left(\beta \right)$; (**b**) $p_{2}\left(\beta \right)$ compared to $D_{p2}\left(\beta \right)$; (**c**) $p_{3}\left(\beta \right)$ compared to $D_{p3}\left(\beta \right)$. The numeric values of $D_{p1}$, $D_{p2}$, and $D_{p3}$ were obtained in the first estimation step. The outliers corresponding to the last FORC at β≈0.92 were not considered in the second estimation step.

Further insight was obtained by comparing the fitted shape variation functions (7)–(9) to datasets $D_{p1}$, $D_{p2}$, and $D_{p3}$, obtained in the first step of estimation. The results are presented in Figure 6, Figure 7 and Figure 8. The comparison for the analyzed NO27 ES is presented in Figure 6. The results for $y_{6,\mathrm{ref}}$ are also included alongside the shape variation functions corresponding to the estimated parameter sets $y_{6}$ and $y_{9}$.

Analogously, the comparison for the analyzed NO32 ES is presented in Figure 7.

Finally, the comparison for the analyzed GO27 ES is shown in Figure 8.

The presented results show that all parameter sets (i.e., $y_{6,\mathrm{ref}}$, $y_{6}$, $y_{9}$) defined shape variation functions that reflected the overall tendencies in $D_{p1}\left(\beta \right)$, $D_{p2}\left(\beta \right)$, and $D_{p3}\left(\beta \right)$ for all analyzed ES samples. The crudest overall approximation of the underlying data was obtained for shape variation functions $p_{3}\left(\beta \right)$, which were restricted by (9) to either a constant or a linear approximation. The main observed difference between the analyzed NO and GO ES samples was the obtained trends in $D_{p3}\left(\beta \right)$, where, for NO ES samples, negative slopes were observed, whereas, for GO ES, the slope was positive.

The estimated parameter sets $y_{6}$ and $y_{9}$ provided better fits to the input data compared to $y_{6,\mathrm{ref}}$, which was sourced from [30]. Overall, the obtained data in the first step of $D_{p1}$, $D_{p2}$, and $D_{p3}$ exhibited some variations in trends with respect to β, including some step-like changes and individual outliers. This inconsistency is evident:

- In Figure 6, at β≈0.45 and β≈0.8;
- In Figure 7, at β≈0.15, β≈0.45, and β≈0.55;
- In Figure 8, at β≈0.92.

A similar behavior was also discovered by other authors, e.g., those presented in Figure 8 in [33]. It is interesting to note that the observed sudden changes were significantly bigger in $D_{p2}$ and $D_{p3}$ compared to $D_{p1}$. Consequently, the impact of the outliers on the shape variation functions $p_{2}\left(\beta \right)$ and $p_{3}\left(\beta \right)$ was significant, whereas the estimated $p_{1}\left(\beta \right)$ was not affected significantly.

The observed changes were analyzed further by applying various optimization methods in the first estimation step. The results demonstrated dependency on the chosen optimization strategy, with the lsqnonlin method yielding consistent outcomes. The sensitivity analysis indicated that the optimization problem (14) incorporating (11) is inherently challenging due to its non-convex nature, which allows for the possibility of multiple local optima. Variations in the results could stem partly from measurement uncertainties, the highly nonlinear and variable shapes of FORCs within the major loop, and the specific formulation of the analyzed model.

### 4.3. Validation Versus Measured FORCs

**Figure 9:**
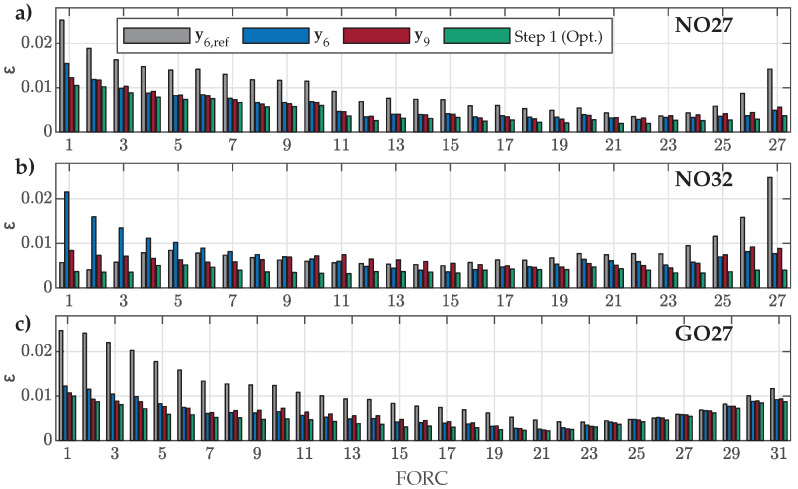
Comparison of calculated NRMS deviation ε between the individual measured and calculated FORCs when applying parameter sets $y_{6,\mathrm{ref}}$, $y_{6}$, or $y_{9}$ for (**a**) NO27 ES, (**b**) NO32 ES, and (**c**) GO27 ES.

The HD model was re-evaluated versus the measured FORCs used for parameter estimation by considering the estimated parameter sets $y_{6}$ and $y_{9}$. The results for the parameter set $y_{6,\mathrm{ref}}$ were added for reference. The goodness of fit, ε, was evaluated again by (16). The results for all three ES types are presented in Figure 9.

The obtained results show that the NRMS deviation ε was generally slightly increased for all FORCs in comparison to the goodness of fit presented in Figure 4. This was a direct consequence of approximating the variations in $D_{p1}$, $D_{p2}$, and $D_{p3}$ with the shape variation functions $p_{1}\left(\beta \right)$, $p_{2}\left(\beta \right)$, and $p_{3}\left(\beta \right)$ in the second step of parameter estimation. However, despite the increase in ε, the obtained values for the estimated sets $y_{6}$ or $y_{9}$ were, for most FORCs, still well under ε=0.01. Both estimated sets, $y_{6}$ or $y_{9}$, performed similarly for all three materials, where $y_{9}$ did not improve the fit over $y_{6}$ significantly, even in the case for the GO27 ES. A visible discrepancy between results for $y_{6}$ or $y_{9}$ was obtained in the case of NO32 ES for FORC Nos. 1–7. This was a direct result of the abrupt change shown in Figure 7, which the shape variation functions could not account for adequately.

Further, the obtained results supported that the parameter set $y_{6,\mathrm{ref}}$ can be adequate for modeling of both analyzed NO ES samples, where the NRMS ε values were, for most of the FORCs, comparable to the results obtained based on $y_{6}$ or $y_{9}$ (for most FORCs, ε was slightly increased, whereas, for some FORCs, ε was even decreased slightly, indicating a better fit). Finally, the results in Figure 9c confirmed that the parameter set $y_{6,\mathrm{ref}}$ is not the best option for modeling magnetization within the analyzed GO27 ES.

**Figure 10:**
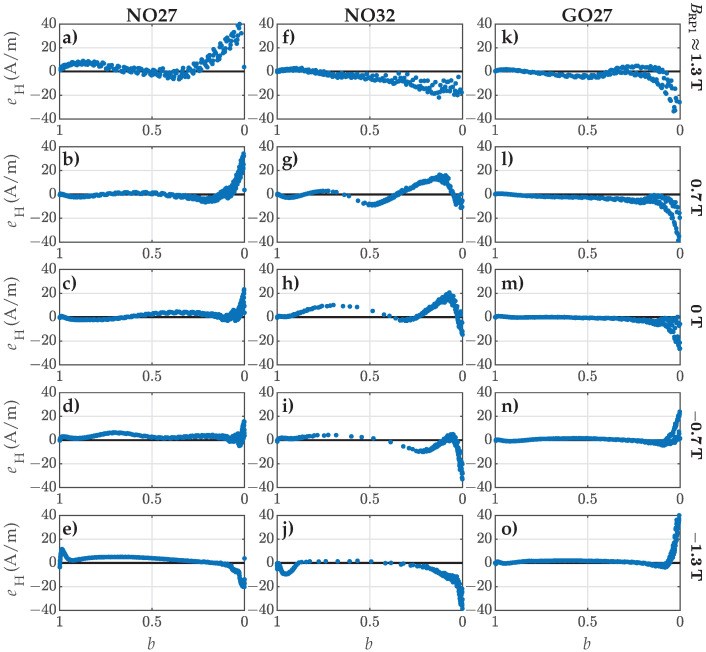
Comparison of the calculated residuals $e_{H}$ along the selected FORCs when considering $p_{1}\left(\beta \right)$, $p_{2}\left(\beta \right)$, and $p_{3}\left(\beta \right)$ obtained in the second step of estimation. The rows correspond to comparable values of $B_{\mathrm{RP}1}$, and the columns correspond to individual ES samples: (**a**–**e**) NO27 ES (FORC No. 1, 7, 14, 21, and 27), (**f**–**j**) NO32 ES (FORC No. 1, 7, 14, 21, and 27), and (**k**–**o**) GO27 ES (FORC No. 3, 9, 16, 23, and 29).

Analogously to the analysis in Section 4.1, we evaluated the residual $e_{H}$ between the measurements and the model. The residuals corresponding to the estimated parameter set, $y_{9}$, are shown in Figure 10.

These results support the conclusions drawn from the NRMS deviation analysis depicted in Figure 9. Additional deviations were introduced to individual FORCs through the consideration of shape variation functions (7)–(9), which modeled trends in $D_{p1}$, $D_{p2}$, and $D_{p3}$. Similarly to the findings in Figure 5, the largest deviations occurred as *B* approached saturation. However, the most significant difference between the results in Figure 5 and Figure 10 was in the approximation of the slopes for individual FORCs. Following the second step of parameter estimation, the goodness of fit concerning the slopes of the modeled FORCs decreased, as evidenced by the increased oscillations in the residuals with respect to *b*.

### 4.4. Validation Versus Measured Symmetric and Offset Minor Loops

**Figure 11:**
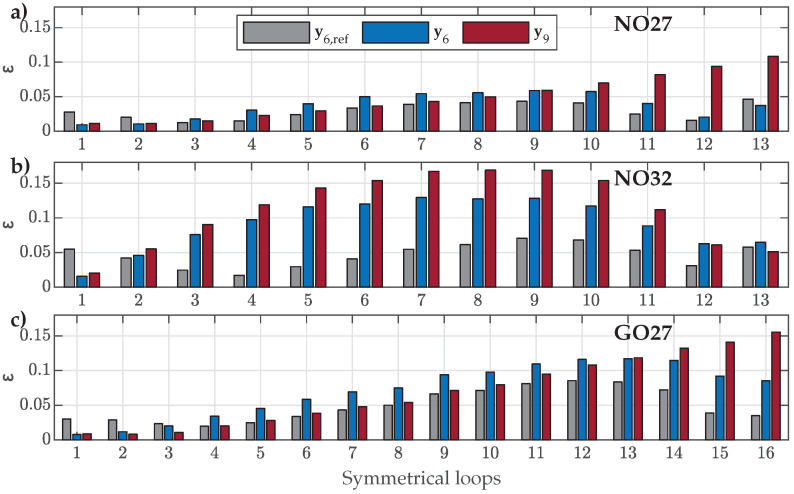
Comparison of the calculated NRMS deviation ε between individual measured and calculated SMLs when applying parameter sets $y_{6,\mathrm{ref}}$, $y_{6}$, or $y_{9}$ for (**a**) NO27 ES, (**b**) NO32 ES, and (**c**) GO27 ES.

The HD model was validated further by comparing the measured and calculated SMLs and OMLs, where all three materials were evaluated with the corresponding estimated parameter sets $y_{6}$ and $y_{9}$. The reference parameter set $y_{6,\mathrm{ref}}$ was included to enhance the presented analysis. First, the goodness of fit was assessed using (16). The results for the SMLs are shown in Figure 11.

The obtained NRMS ε values are significantly increased compared to the previous FORC-based evaluation. However, they are, for the majority of SMLs, well under ε=0.15. Overall, all three parameter sets resulted in comparable ε. The biggest deviations were observed when applying $y_{9}$, especially for SMLs with small amplitudes in the cases of NO27 ES and GO27 ES and for SMLs with medium amplitudes in the case of NO32 ES. The best overall goodness of fit was achieved by considering $y_{6,\mathrm{ref}}$, even in the case of GO27 ES. It is interesting to note that $y_{6,\mathrm{ref}}$ resulted in the worst performance when analyzing FORCs for GO27 ES, as presented in Figure 9c. These results highlight the complex nature of static hysteretic magnetization, where generalizing the curve shapes of different orders and positions within the major loop is highly challenging. The generalization in the analyzed model assumes that the family of RCs within all possible outer loops is based on $p_{1}\left(\beta \right)$, $p_{2}\left(\beta \right)$, and $p_{3}\left(\beta \right)$. The SMLs were reconstructed based on the FORCs in the analyzed case. In contrast to this, the parameter set $y_{6,\mathrm{ref}}$ was estimated by the authors using SMLs. This distinction may explain why the goodness of fit was better in the case of applying $y_{6,\mathrm{ref}}$ for the majority of SMLs across all three analyzed ES samples.

**Figure 12:**
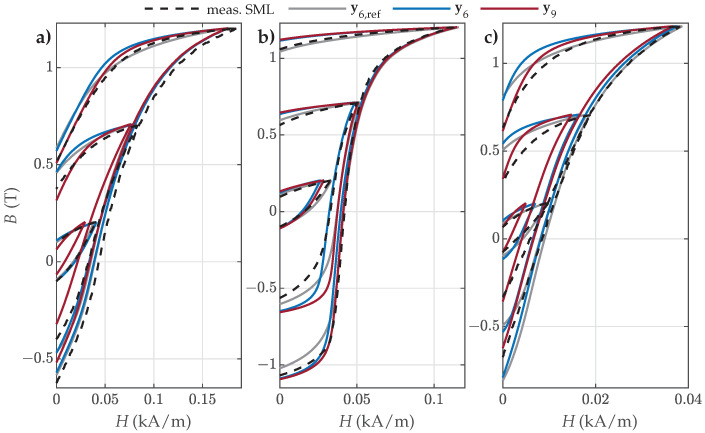
Comparison of the measured and calculated SMLs when applying parameter sets $y_{6,\mathrm{ref}}$, $y_{6}$, or $y_{9}$ for (**a**) NO27 ES (SML Nos. 2, 7, and 12), (**b**) NO32 ES (SML Nos. 2, 7, and 12), and (**c**) GO27 ES (SML Nos. 5, 10, and 15).

Finally, the comparison between the selected measured and reconstructed SMLs is presented in Figure 12. The obtained results show that the predicted SMLs varied in accuracy, but overall, they described the measured SMLs more or less well. The best overall fit was obtained by applying the reference parameter set $y_{6,\mathrm{ref}}$. The visual comparison in Figure 12 confirmed the results presented in Figure 11:

- For NO27 ES, all three parameter sets performed comparably well, except that applying $y_{9}$ resulted in a bigger deviation for SMLs with small amplitudes, where the predicted SMLs were significantly narrower and steeper.
- For NO32 ES, all three parameter sets exhibited comparable performance. However, applying $y_{9}$ led to greater deviations for SMLs with medium amplitudes, where the predicted SMLs were wider.
- For GO27 ES, all three parameter sets performed comparably well, except that applying $y_{9}$ resulted in a bigger deviation for SMLs with small amplitudes, where the predicted SMLs were significantly steeper.

**Figure 13:**
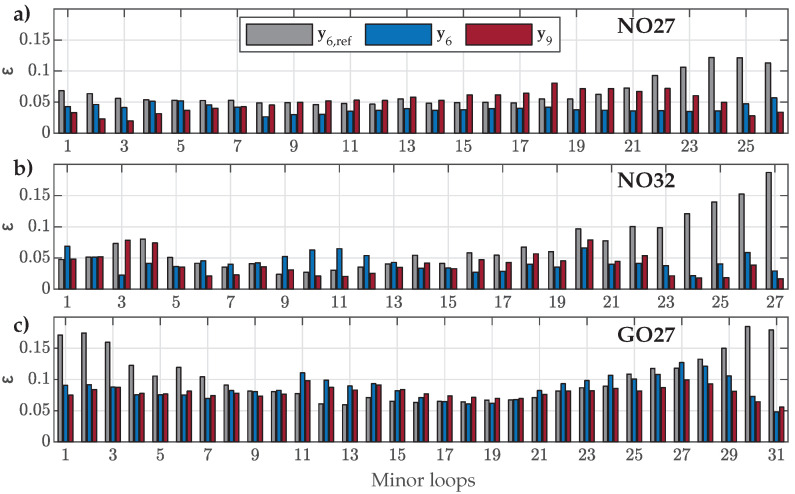
Comparison of the calculated NRMS ε values between individual measured and calculated OMLs when applying parameter sets $y_{6,\mathrm{ref}}$, $y_{6}$, or $y_{9}$ for (**a**) NO27 ES, (**b**) NO32 ES, and (**c**) GO27 ES.

Finally, validation was also performed on measured OMLs along the major loop, consisting of FORCs and SORCs. The goodness of fit is presented in Figure 13.

The results are comparable to the goodness of fit of SMLs. Almost all the evaluated NRMS deviations ε were well below ε=0.1, and for both NO ES samples, many were under ε=0.05. The most consistent results were obtained for NO ES. In the case of the analyzed GO27 ES, applying the recommended reference set $y_{6,\mathrm{ref}}$ resulted in the worst goodness of fit, especially for the OMLs positioned around the knee regions of the major loop.

**Figure 14:**
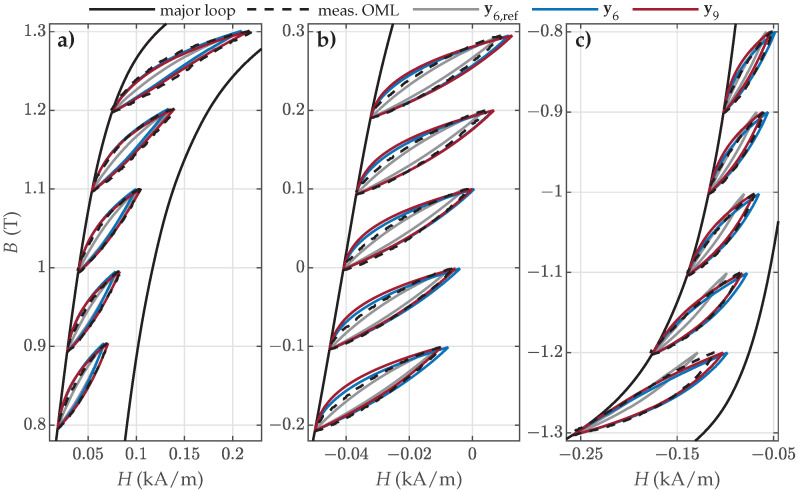
Comparison of the measured and calculated OMLs when applying parameter sets $y_{6,\mathrm{ref}}$, $y_{6}$, or $y_{9}$ for modeling of NO27 ES: (**a**) OML Nos. 1–5; (**b**) OML Nos. 11–15; and (**c**) OML Nos. 22–26.

**Figure 15:**
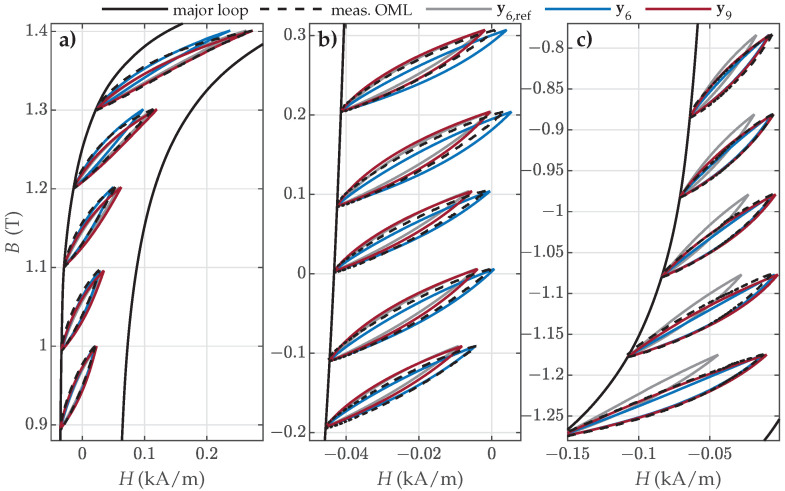
Comparison of the measured and calculated OMLs when applying parameter sets $y_{6,\mathrm{ref}}$, $y_{6}$, or $y_{9}$ for the modeling of NO32 ES: (**a**) OML Nos. 1–5, (**b**) OML Nos. 12–16, and (**c**) OML Nos. 23–27.

**Figure 16:**
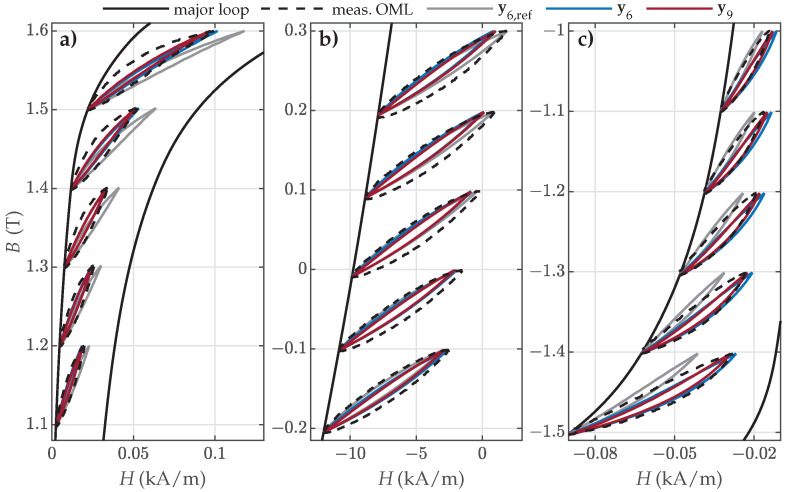
Comparison of the measured and calculated OMLs when applying parameter sets $y_{6,\mathrm{ref}}$, $y_{6}$, or $y_{9}$ for the modeling of GO27 ES: (**a**) OML Nos. 1–5, (**b**) OML Nos. 14–18, and (**c**) OML Nos. 27–31.

The visual comparison between the selected measured and calculated OMLs is presented in Figure 14, Figure 15 and Figure 16. The results for NO27 ES are presented in Figure 14.

The visual comparison revealed that the predicted OMLs were very similar in the upper part of the descending major loop branch (Figure 14a) for all three parameter sets (i.e., for OML Nos. 1 to 5). The OMLs in the middle part of the descending major loop branch (Figure 14b) were best approximated with the parameter set $y_{6}$, whereas the OMLs in the lower part of the descending major loop branch (Figure 14c) were significantly poorer (too narrow and too steep) when approximated with the reference set $y_{6,\mathrm{ref}}$. Overall, the estimated sets $y_{6}$ and $y_{9}$ outperformed the reference set $y_{6,\mathrm{ref}}$, with $y_{6}$ providing the best predictions.

Analogous results were obtained in the case of NO32 ES (Figure 15).

The predicted OMLs were very similar in the upper and middle parts of the descending major loop branch (Figure 15a,b) for all three parameter sets. The OMLs in the lower part of the descending major loop branch (Figure 15c) were significantly worse when approximated with the reference set $y_{6,\mathrm{ref}}$. Overall, the estimated sets $y_{6}$ and $y_{9}$ outperformed the reference set $y_{6,\mathrm{ref}}$.

In the case of GO27 ES, the accuracy of the obtained results was decreased overall compared to NO ES, as presented in Figure 16. All predicted OMLs were generally too narrow; however, the estimated sets $y_{6}$ and $y_{9}$ performed notably better compared to $y_{6,\mathrm{ref}}$, especially in the upper and lower parts of the descending major loop branch, as presented in Figure 16a,c. It is interesting to note that the results corresponding to all three datasets were very similar in the middle part of the descending branch, as presented in Figure 16b.

## 5. Discussion

### 5.1. Accuracy

Overall, the performed validation analysis confirmed that the analyzed HD hysteresis model can be parameterized based on the proposed two-step approach. The predicted SMLs, as well as OMLs, were reasonably accurate for all three analyzed ES samples. In the case of NO ES, the estimated parameter set $y_{6}$ resulted in comparable or better predictions overall compared to parameter set $y_{9}$, confirming the adequacy of modeling the shape variation functions $p_{1}\left(\Delta B_{\mathrm{rev}},\beta \right)$, $p_{2}\left(\beta \right)$, and $p_{3}\left(\beta \right)$ with six parameters. It is important to note that there is no significant difference in the computational cost and time of the ZM model when considering six or nine parameters. Furthermore, the results confirmed good prediction accuracy when $y_{6,\mathrm{ref}}$ was applied, where $y_{6,\mathrm{ref}}$ resulted in better predictions for SMLs and $y_{6}$ resulted in better predictions for OMLs. The increased accuracy for the specific families of magnetization curves was most likely rooted in the fundamental curves used in the individual estimation approaches.

The results highlighted further the increased difficulty when predicting magnetization curves within the analyzed GO27 ES. GO ES samples have, in general, much steeper magnetization curves with higher nonlinearity, resulting in less accurate fitting. The results additionally showed that the differences between applying $y_{6}$ and $y_{9}$ are small, where the three additional parameters within the shape variation functions $p_{1}\left(\Delta B_{\mathrm{rev}},\beta \right)$, $p_{2}\left(\beta \right)$, and $p_{3}\left(\beta \right)$ did not necessarily improve predictions. To improve the accuracy, both the shape variation functions, as well as the main model (Equation (5)) should be adjusted.

### 5.2. Computational Complexity

One of the advantages of the presented two-step estimation approach is the reduced computational complexity compared to the direct estimation approach. Saturation is considered as the HI initial condition of the presented HD model, as presented in Section 2.6. Consequently, all inner curves and loops (including SMLs) can only be obtained through adequate initial demagnetization. Such a demagnetization routine builds up the memory of the model, determining the shapes of the inner loops, and is parameter-dependent. Therefore, demagnetization must be performed in each optimization iteration. To achieve adequate memory content, demagnetization with many cycles (i.e., a small demagnetization step) is necessary, increasing the computational time and effort within the optimization procedure.

A single demagnetization involving 100 cycles took approximately 50 s to complete in the Matlab-implemented version of the ZM HD model. The subsequent evaluation of the cost function contributed minimally to the overall duration of a single iteration (around 2 s). Considering the total time per iteration multiplied by the number of optimization iterations, direct estimation proved to be a time-intensive process, taking several hours to complete. Conversely, the two-step estimation approach was significantly more efficient. In the first step, fitting the main model equation directly to the measured FORCs required only about 1.5 s for all the FORCs combined. The second step, which involved fitting the parameter dependencies, had a negligible impact on the overall time. In summary, two-step estimation can be performed within seconds, whereas direct estimation requires hours.

### 5.3. Physical Interpretation of the Parameters

The analyzed ZM HD hysteresis model is a phenomenological model. Therefore, there is no relation to the underlying physics, and it is even challenging to indicate qualitatively which parameters could resemble the effects of specific physical mechanisms. A tentative indication can be made by comparing the effects of changing the parameters to those in a more physically derived hysteresis model, such as the well-known Jiles–Atherton (JA) model, where adding such an indication was requested by reviewers. The JA model has five parameters: the magnetization saturation parameter $M_{s}$, the shape parameter *a*, the mean-field parameter α, the domain wall-pinning parameter *k*, and the domain wall-flexing parameter *c*. The impacts of these parameters on the loop’s shape are presented in [18]. In contrast, the ZM model described by (5) only has three fundamental parameters: $p_{1}$ governs the rate of decay of the fast (exponential) component of ΔH, $p_{3}$ governs the rate of decay of the slow component of ΔH, and $p_{2}$ balances both discussed components of ΔH. The impact of the parameter values on the constructed magnetization curves is presented in [4]. By comparing the impacts of the parameter values in both models, a tentative indication can be made in that the JA loop shape parameters are considered implicitly in the ZM model’s major loop shape, whereas the domain-related parameters *c* and *k* are related to parameters $p_{1}$, $p_{2}$, and $p_{3}$. However, to find stronger correlations, a detailed analysis is required, which is beyond the scope of the presented research.

### 5.4. Multiphysics Extension

It is worthwhile to note that the presented ZM model could be enhanced further by implementing temperature- or stress-dependent parameters. Stress-dependent behavior could be implemented similarly to the approach presented in [16], where a stress term could be introduced to parameters $p_{1}$, $p_{2}$, and $p_{3}$ (in a manner similar to their dependence on beta). Additionally, a shape modifier for the major loop should be introduced.

## 6. Conclusions

The analyzed two-step approach represents a fundamental method for estimating the parameters of the presented HD ZM hysteresis model. This approach leverages the core principle of the model: the construction of HD magnetization curves within the so-called outer loops. By examining the most basic HD magnetization curves, specifically the family of measured FORCs, the parameters of the HD hysteresis model can be estimated using only the corresponding measured major loop branches. This estimation assumes that the shape variation functions $p_{1}\left(\Delta B_{\mathrm{rev}},\beta \right)$, $p_{2}\left(\beta \right)$, and $p_{3}\left(\beta \right)$ can be generalized to describe shape variations across all possible outer loops within the major loop.

The presented approach offers notable advantages, including computational efficiency and the ability to systematically analyze trends in the shape variations of curves during the first estimation step. Such an analysis would not be feasible if the model was fitted directly, as parameter sets with six or nine parameters would be estimated without this intermediate step. However, a limitation of the analyzed approach is that it necessitates measuring FORCs corresponding to the different relative positions of RPs, as described by β, within the major loop. Such RCs are not part of the standardized measurements conducted for NO and GO ESs, and they are therefore often unavailable.

The results of the systematic analysis indicate that obtaining optimal values for $p_{1}$, $p_{2}$, and $p_{3}$ can be challenging. The optimization problem is likely non-convex, making it sensitive to the chosen method, and it is influenced by measurement uncertainties and nonlinear variations between individual loop shapes within the family of FORCs. The estimation of $p_{2}$ and $p_{3}$ was particularly sensitive for NO ESs, whereas the estimation for the analyzed GO27 ES showed lower sensitivity.

The fitting of shape variation functions $p_{1}\left(\Delta B_{\mathrm{rev}},\beta \right)$, $p_{2}\left(\beta \right)$, and $p_{3}\left(\beta \right)$ in the second step was influenced by outliers, which affected the model’s accuracy during the validation step. Nonetheless, the analysis confirmed that the recommended parameter set $y_{6,\mathrm{ref}}$ from [4] is reasonably accurate for a range of NO ES samples, and it might be reasonable even for the undemanding modeling of some GO steel types. Its accuracy is particularly notable when predicting SMLs, as $y_{6,\mathrm{ref}}$ was estimated using measured data that included such loops.

Furthermore, the accuracy of the estimated parameter sets $y_{6}$ and $y_{9}$ is comparable, supporting the validity of the generalization assumption for curves across different outer loops. The accuracy of individual RCs improved when the presented two-step identification process was applied. Additionally, the results demonstrated that using the shape variation functions with $y_{6}$ offers comparable or even superior accuracy compared to those with $y_{9}$. In the former case, the shape variation function $p_{1}\left(\Delta B_{\mathrm{rev}},\beta \right)$ can be represented effectively by a polynomial, $p_{2}\left(\beta \right)$ exhibits a decreasing linear trend, and $p_{3}\left(\beta \right)$ can be well approximated by a constant value.

Future work will include a systematic investigation of the impact of experimental data distribution, quality, and quantity on the discussed parameter estimation, focusing on the first step of the estimation. The analysis will include the implementation of various numerical methods, and it will focus on obtaining smoother trends in the obtained data. Additionally, we plan to extend the ZM model into its vectorized form and investigate the parameter estimation methods of the vectorized model.

## Figures and Tables

**Table 1:** Comparison of measured magnetization curves and hysteresis loops for NO27, NO32, and GO27 ES samples.

	$B_{\mathrm{sat}}$	$H_{\mathrm{sat}}$	$N_{\mathrm{SML}}$	$N_{\mathrm{FORC}}$	$N_{\mathrm{OML}}$
	(T)	($\frac{A}{m}$)			
NO27 ES	1.469	1010.1	13	27	26
NO32 ES	1.516	1013.4	13	27	27
GO27 ES	1.807	1001.8	16	31	31

**Table 2:** Comparison of the estimated parameters for NO27, NO32, and GO27 ES grades. Each analyzed ES was fitted by both 6 and 9 parameters.

	NO27 ES		NO32 ES		GO27 ES	
	$y_{6}$	$y_{9}$	$y_{6}$	$y_{9}$	$y_{6}$	$y_{9}$
$y_{1}$	15.81	15.81	3.794	3.794	10.54	10.54
$y_{2}$	18.2	18.2	84.8	84.8	−17.65	−17.65
$y_{3}$	−112.9	−112.9	−231.8	−231.8	11.73	11.73
$y_{4}$	102.3	102.3	167.5	167.5	14.77	14.77
$y_{5}$	0.6946	1.926	0.4877	−0.027	0.4869	1.38
$y_{6}$	/	−3.714	/	2.727	/	−2.786
$y_{7}$	/	2.613	/	−2.645	/	2.024
$y_{8}$	1.4	1.993	0.2733	0.5743	0.129	1.9 × 10^−7^
$y_{9}$	/	−1.171	/	−0.5743	/	0.3355

## Data Availability

The original contributions presented in this study are included in the article. Further inquiries can be directed to the corresponding author.

## Abbreviations

The following abbreviations are used in this manuscript:

ES	Electrical steel;
FORC	First-order reversal curve;
GO	Grain oriented;
HD	History dependent;
HI	History independent;
JA	Jiles–Atherton;
NO	Non-oriented;
NRMS	Normalized root mean square;
OML	Offset minor loop;
RC	Reversal curve;
RP	Reversal point;
SML	Symmetric minor loop;
SORC	Second-order reversal curve;
ZM	Zirka–Moroz.
